# Massachusetts Veterinary Association

**Published:** 1895-12

**Authors:** Howard P. Rogers

**Affiliations:** Secretary


Secretary.

### MASSACHUSETTS VETERINARY ASSOCIATION.

THE regular monthly meeting was held at 19 Boylston Place, Boston, September 25, 1895, at 8 P.M., the President, Dr. J. M. Parker, in the chair. A large number of the members were present. Minutes of the last meeting were read and accepted. Dr. F. H. Osgood, as one of the delegates to the U. S. V. M. Association reported the proceedings of the annual meeting at

Des Moines. The advisability of employing a stenographer was discussed, and the matter finally left in the hands of a committee consisting of Drs. L. H. Howard and H. P. Rogers. On motion of Dr. Osgood, Drs. Beckett, Blackwood, and Winchester were appointed a committee to draw up resolutions on the death of Dr. Williamson Bryden. A large number of interesting cases were reported, the discussion of which lasted until a late hour, and making it one of the most interesting meetings we have had for some time, the everlasting subject of tuberculosis not being mentioned.

The regular monthly meeting was held at 19 Boylston Place, Boston, Wednesday evening, October 23, 1895, Dr. Parker in the chair. There was a good attendance. Minutes of last meeting were read and accepted. Dr. Howard reported for the Executive Committee that the Secretary be instructed to send notices that the by-laws will be enforced where members do not pay their dues before January 1, 1896. Dr. Howard also reported for special committee on stenographer, after which a motion was made and carried that Dr. Howard be given full power to employ a stenographer for our next meeting.

Dr. Beckett reported for the special committee on resolutions on the death of Dr. Bryden (Drs. Beckett, Blackwood, and Winchester) as follows: "Whereas, it has pleased the Almighty in His divine providence to remove from our midst our esteemed colleague, Williamson Bryden; and, whereas, the intimate relation and business intercourse with him have been most pleasant, it makes it befitting that we publicly record our appreciation of him; therefore, be it resolved that in the loss of Dr. Williamson Bryden we lose a friend and valued member of our Association and profession; therefore, be it resolved that the deep sympathy of this Association be extended to his relatives and friends; and be it further resolved that a copy of these resolutions be forwarded to his relatives, spread upon our records, and published in the veterinary journals."

A number of interesting cases were reported and discussed. Dr. A. Shannon, of Malden, applied for membership. Drs. Beckett, Parker, Labaw, and Lewis promised papers for future meetings.

HOWARD P. ROGERS,
Secretary.

